# The Genealogical Population Dynamics of HIV-1 in a Large Transmission Chain: Bridging within and among Host Evolutionary Rates

**DOI:** 10.1371/journal.pcbi.1003505

**Published:** 2014-04-03

**Authors:** Bram Vrancken, Andrew Rambaut, Marc A. Suchard, Alexei Drummond, Guy Baele, Inge Derdelinckx, Eric Van Wijngaerden, Anne-Mieke Vandamme, Kristel Van Laethem, Philippe Lemey

**Affiliations:** 1 Department of Microbiology and Immunology, Rega Institute, KU Leuven, Leuven, Belgium; 2 Institute of Evolutionary Biology, University of Edinburgh, Edinburgh, United Kingdom; 3 Fogarty International Center, National Institutes of Health, Bethesda, Maryland, United States of America; 4 Department of Biomathematics, David Geffen School of Medicine at UCLA, University of California, Los Angeles, Los Angeles, California, United States of America; 5 Department of Human Genetics, David Geffen School of Medicine at UCLA, University of California, Los Angeles, Los Angeles, California, United States of America; 6 Department of Biostatistics, UCLA Fielding School of Public Health, University of California, Los Angeles Los Angeles, California, United States of America; 7 Allan Wilson Centre for Molecular Ecology and Evolution, University of Auckland, Auckland, New Zealand; 8 University Hospitals Leuven, Leuven, Belgium; 9 Centro de Malária e Outras Doenças Tropicais Instituto de Higiene e Medicina Tropical and Unidade de Microbiologia, Universidade Nova de Lisboa, Lisboa, Portugal; Imperial College London, United Kingdom

## Abstract

Transmission lies at the interface of human immunodeficiency virus type 1 (HIV-1) evolution within and among hosts and separates distinct selective pressures that impose differences in both the mode of diversification and the tempo of evolution. In the absence of comprehensive direct comparative analyses of the evolutionary processes at different biological scales, our understanding of how fast within-host HIV-1 evolutionary rates translate to lower rates at the between host level remains incomplete. Here, we address this by analyzing *pol* and *env* data from a large HIV-1 subtype C transmission chain for which both the timing and the direction is known for most transmission events. To this purpose, we develop a new transmission model in a Bayesian genealogical inference framework and demonstrate how to constrain the viral evolutionary history to be compatible with the transmission history while simultaneously inferring the within-host evolutionary and population dynamics. We show that accommodating a transmission bottleneck affords the best fit our data, but the sparse within-host HIV-1 sampling prevents accurate quantification of the concomitant loss in genetic diversity. We draw inference under the transmission model to estimate HIV-1 evolutionary rates among epidemiologically-related patients and demonstrate that they lie in between fast intra-host rates and lower rates among epidemiologically unrelated individuals infected with HIV subtype C. Using a new molecular clock approach, we quantify and find support for a lower evolutionary rate along branches that accommodate a transmission event or branches that represent the entire backbone of transmitted lineages in our transmission history. Finally, we recover the rate differences at the different biological scales for both synonymous and non-synonymous substitution rates, which is only compatible with the ‘store and retrieve’ hypothesis positing that viruses stored early in latently infected cells preferentially transmit or establish new infections upon reactivation.

## Author Summary

Since its discovery three decades ago, the HIV epidemic has unfolded into one of the most devastating pandemics in human history. When HIV replication cannot be completely inhibited, the fast-evolving retrovirus continuously evades intra-host immune and drug selective pressure, but diversifies according to more neutral epidemiological dynamics at the interhost level. Limited evidence suggests that the virus may evolve faster in a single host than in a population of hosts, and various hypotheses have been put forward to explain this phenomenon. Here, we develop a new computational approach aimed at integrating host transmission information with pathogen genealogical reconstructions. We apply this approach to comprehensive sequence data sets sampled from a large HIV-1 subtype C transmission chain, and in addition to providing several insights into the reconstruction of HIV-1 transmissions histories and its associated population dynamics, we find that transmission decreases the HIV-1 evolutionary rate. The fact that we also identify this decline for substitutions that do not alter amino acid substitutions provides evidence against hypotheses that invoke selection forces. Instead, our findings support earlier reports that new infections start preferentially with less evolved variants, which may be stored in latently infected cells, and this may vary among different HIV-1 subtypes.

## Introduction

HIV evolutionary analyses generally focus on either within-host dynamics or on among-host epidemiological processes [1]. The rapid evolutionary rate of HIV allows the virus to accumulate significant sequence divergence over the time course of a single infection, ensuring that within-host HIV populations can escape both considerable immune and drug selective pressure. Across multiple infections, however, these selective dynamics and within-host evolutionary arms race do not appear to strongly impact the mode of HIV diversification, as multiple co-circulating lineages generally reflect more neutral epidemiological dynamics [2]. The mechanisms involved in HIV transmission are key to this distinction and they have received a great deal of attention due to their importance for the design of preventive strategies (e.g. [3]). Although transmission generally imposes a strong bottleneck on HIV within-host populations [4], [5], no clear phenotypic constraints appear to act on transmission apart, perhaps, from co-receptor usage, and a multitude of viral phenotypical aspects are only loosely associated with enhanced transmission [6].

In addition to studies characterizing viral founder populations, phylogenetic studies also take great interest in sequence data sampled across multiple infections. Molecular phylogenetics represents a popular approach to elucidate transmission links in a wide variety of situations, including nosocomial transmission from health care workers [7], [8], mother-to-child transmission [9], sexual transmission [10], [11], parenteral transmission [12] and even criminal transmission [13], [14]. Its use as a forensic tool has led to a critical appraisal of viral phylogenetics e.g. [15], [16], and in this respect, known transmission histories may provide valuable data to evaluate the performance of the evolutionary reconstruction methods. By comparing inferred clustering patterns with the known phylogenetic relationships in a Swedish transmission chain, Leitner *et al.* [17] were the first to demonstrate that phylogenetic estimates were generally consistent with the transmission history, provided the evolutionary model accounts for rate variation. A more recent analysis of an HIV transmission cluster involving 9 patients also presented phylogenetic reconstructions that were largely compatible with the known transmission history, except for one particular transmission link that appeared to be confounded by multi-drug resistance patterns in the *pol* gene [18]. Whereas Leitner *et al.* examined topological differences between a single viral and transmission tree, the more recent study took a somewhat different perspective on compatibility and examined whether any conflict arises when attempting to superimpose the host transmission history onto the viral phylogeny. This was motivated by the fact that different viral evolutionary trajectories can be embedded within a particular host transmission tree, akin to gene trees and their containing species trees [19].

In addition to confirming transmission links, the question has also been raised to what extent transmission direction and even transmission times can be ascertained through phylogenetic approaches. The former may be inferred through paraphyletic clustering of the source viruses with respect to those of the recipient, which requires adequate sampling of the viral diversity within both source and recipient [20] or samples from the source both before and after transmission [18]. To explore the temporal dimension of viral transmission, phylogenetic trees need to be calibrated in time units. This is accommodated by the incorporation of molecular clock models in phylogenetic inference and has proven useful to test hypotheses on HIV-1 and HCV transmission [21]. Applications to next-generation sequencing data have further exploited time-measured trees to provide genetic estimates of dates of HIV infection [22], although it needs to be acknowledged that - even when a bottleneck can generally be assumed - the transmission may have occurred anywhere between the divergence from the source and the most recent common ancestor of the recipient viruses [23].

The ability to estimate divergence times and evolutionary rates from time-stamped sequence data has provided a historical perspective on the emergence of different viruses (e.g. [24]) and resulted in detailed investigations into the tempo of evolution at different evolutionary scales [25], [26]. Such studies also led to the suggestion that HIV evolutionary rates may be higher within hosts compared to among hosts. Although few attempts have been made to quantify such differences, different hypotheses have been put forward to explain a potential rate discrepancy [27] and modeling efforts have been undertaken to examine them [28]. From an evolutionary biology perspective, it is difficult to explain such differences in the tempo of evolution at the different scales, and similar to differences in the mode of phylogenetic diversification, they may be dependent on how transmission is linked to within-host evolutionary dynamics. A rate mismatch may arise from the preferential transmission of stored virus, which will be ancestral to the currently circulating diversity in the source patient, and this will result in the accumulation of fewer substitutions between hosts (‘store and retrieve’) [28], [29]. This is in line with a recent phylogenetic study that provided a genome-wide quantification of rate differences within and among-host, and although based on limited within-host data, the consistently-elevated rates across the entire genome seem to support the hypothesis that HIV strains that are less adapted to the host have an advantage during transmission [30]. Alternatively, it has been proposed the within-host adaptive process will have little impact on between host evolutionary rate estimates because many transmissions will occur early in infection before the host mounts effective immune responses (‘stage-specific selection’) [27], [31]. Finally, Herbeck *et al.* [32] explain the rate mismatch by invoking frequent reversion of adaptive mutations when virus enters a new host mounting different immune responses (‘adapt and revert’).

Here, we present a new Bayesian genealogical inference approach that reconstructs within-host viral evolution and population dynamics for different individuals linked in a transmission cluster. At the core of this approach lies a transmission model that requires viral genealogies to be compatible with a timed history of transmission events from a coalescent perspective. Specifically, the model constrains the coalescent time for the source and recipient viral population to be older than the transmission event and assumes a host transition in the viral genealogy upon transmission. This approach (i) further relaxes requirements for topological compatibility between host and viral evolutionary history, (ii) makes no assumption about transmission bottlenecks, and (iii) makes more explicit use of the temporal dimension in viral evolutionary reconstructions from serially-sampled data. Importantly, the transmission constraints and associated parameterization of transmission times allow partitioning of the viral genealogy into patient-specific evolutionary trajectories, each informing the parameters of an overall within-host demographic model. We apply this approach to new clonal HIV-1 subtype C data from a previously-described [18], but extended heterosexual transmission chain. Before applying the model, we test molecular clock models and evaluate the compatibility of the viral evolutionary history with the transmission model constraints. We subsequently explore the model's ability to estimate transmission bottlenecks and transmission times, and use it to quantify evolutionary rates at the interface of within and among-host HIV evolution. Our analyses clearly indicate that transmission decreases HIV-1 evolutionary rates, and since this is the case for both synonymous and non-synonymous substitutions, the findings are consistent with the hypothesis of preferential transmission of ancestral virus.

## Results

### Sequence data

We amplified and sequenced partial *pol* and *env* regions for multiple clones from 11 patients in a previously-studied HIV-1 subtype C transmission chain [18]. Our clonal sampling includes sequences from additional time points for six out of nine previously described patients as well as sequences from two newly identified patients in the transmission cluster (K and L; Figure S1). Written informed consent was obtained from each patient [18]. Patient A and B represent the earliest infected patients in this cluster, but the time and direction of transmission between these two patients has not been clearly established. For the other transmission events, patient interviews and clinical data were able to demarcate a relatively narrow time interval for transmission (see Table S1). Table S2 lists the sampling date, the number of *pol* and *env* clonal sequences obtained for each sample and the sample viral load (if known).

### Bayesian genealogical inference

#### Testing molecular clock models

Because the compatibility constraints we introduce as part of the transmission model condition on divergence times for the viral lineages, we first determined the most appropriate molecular clock model using a standard Bayesian genealogical reconstruction with a flexible coalescent prior (the Skyride model, [33]). We compared strict and relaxed molecular clock models [34] using log marginal likelihoods estimated by recent implementations of path sampling (PS) and stepping-stone (SS) sampling [35], [36] (Table S3). For both the *pol* and *env* data sets, uncorrelated relaxed molecular clocks provide a better model-fit compared to the strict molecular clock. Consistent with general findings in previous analyses [36], a model that considers a lognormal distribution to model rate variation among lineages consistently outperforms a model using an exponential distribution. We therefore performed all further analysis using this relaxed clock parameterization.

#### Compatibility between viral evolutionary history and transmission history

**Figure 1:**
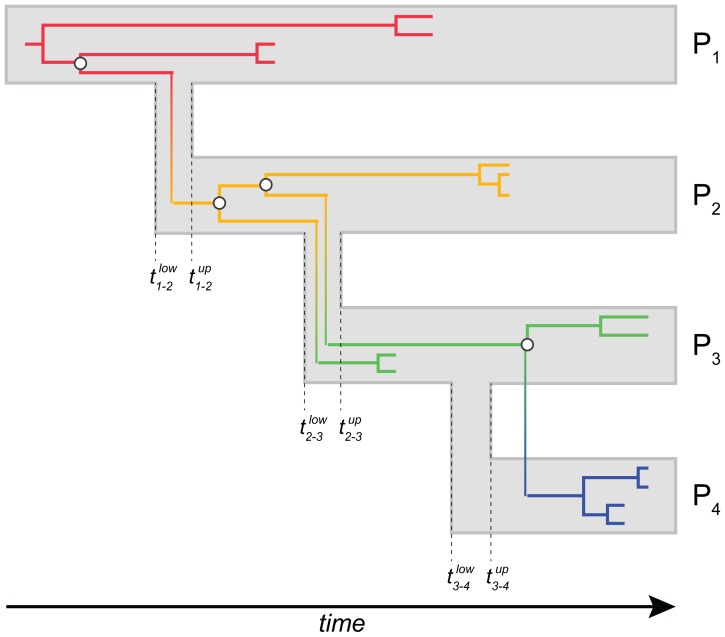
A hypothetical transmission chain and viral genealogy for 4 patients. For each transmission event, we show an upper and lower boundary for the transmission event. The viral lineages within each each patient are represented by a particular branch color, while the transmission-associated host transitions in the viral genealogy are depicted using a color gradient. Whereas the viral genealogy is compatible with transmission from P1 to P2 and P2 to P3, independent of the number of lineages transmitted, the most recent common ancestor for the P3 and P4 lineages is too recent to be compatible with the respective transmission event.

Before assuming coalescent compatibility under the transmission model, we investigated to what extent the viral genealogy meets these constraints using a standard tree prior. The compatibility constraints constitute an essential part of the transmission model and enforce source and recipient lineages to coalesce before transmission time while superimposing the source-recipient transition onto the relevant viral lineages to ensure that the genealogy follows the known chain of transmission events (as illustrated in Figure 1; cfr. Methods). We performed a Bayesian genealogical inference using a flexible tree prior and summarize, for each transmission event, the frequency by which the relevant viral coalescent patterns are indeed compatible with the timed order of transmissions with Bayesian inference using Markov chain Monte Carlo (MCMC) analysis. These posterior compatibility probabilities, together with molecular clock and divergence time estimates are listed in Table 1 for both *pol* and *env*.

**Table 1:** Molecular clock estimates and compatibility probabilities for the *pol* and *env* sequences.

	*pol*	*pol* excluding DRMs^2^	*env*
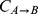 / 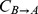 1	0.98/0,98	0.94/0.94	0/0
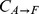	1.00	1.00	1.00
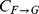	1.00	1.00	1.00
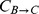	1.00	1.00	0.99
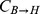	0	0.30	1.00
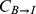	0.78	0.36	0.99
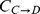	0.01	0.18	0.18
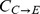	1.00	1.00	1.00
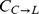	1.00	1.00	1.00
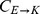	1.00	1.00	1.00
evolutionary rate (95% HPD)	4.75 (3.98–5.54)	4.39 (3.67–5.11)	7.40 (6.36–8.44)
coefficient of variation (95% HPD)	0.53 (0.40–0.66)	0.47 (0.33–0.61)	0.55 (0.42–0.67)
tMRCA (95% HPD)	16.95 (16.53–17.44)	16.99 (16.54–17.49)	16.45 (16.10–16.80)

Compatibility is expressed as the proportion of trees in the posterior sample that is compatible with the indicated transmission event after removal of 10% as burn-in. The mean evolutionary rate and highest posterior density (HPD) intervals are expressed as the number of nucleotide substitutions (10^−3^) per site per year. The coefficient of variation represents the scaled variance in evolutionary rate among lineages. The time to the most recent common ancestor (tMRCA) represents the time since the most recent sampling date (24/03/2006), and is expressed in years.

1 The percentage of sampled genealogies with an entirely compatible coalescent history (C) are listed for the transmission histories assuming A as the original source (A
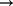
B) as well as B as the original source (B
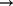
A).

2 DRMs = drug resistance mutations. See [18] for an overview of the removed positions.

**Figure 2:**
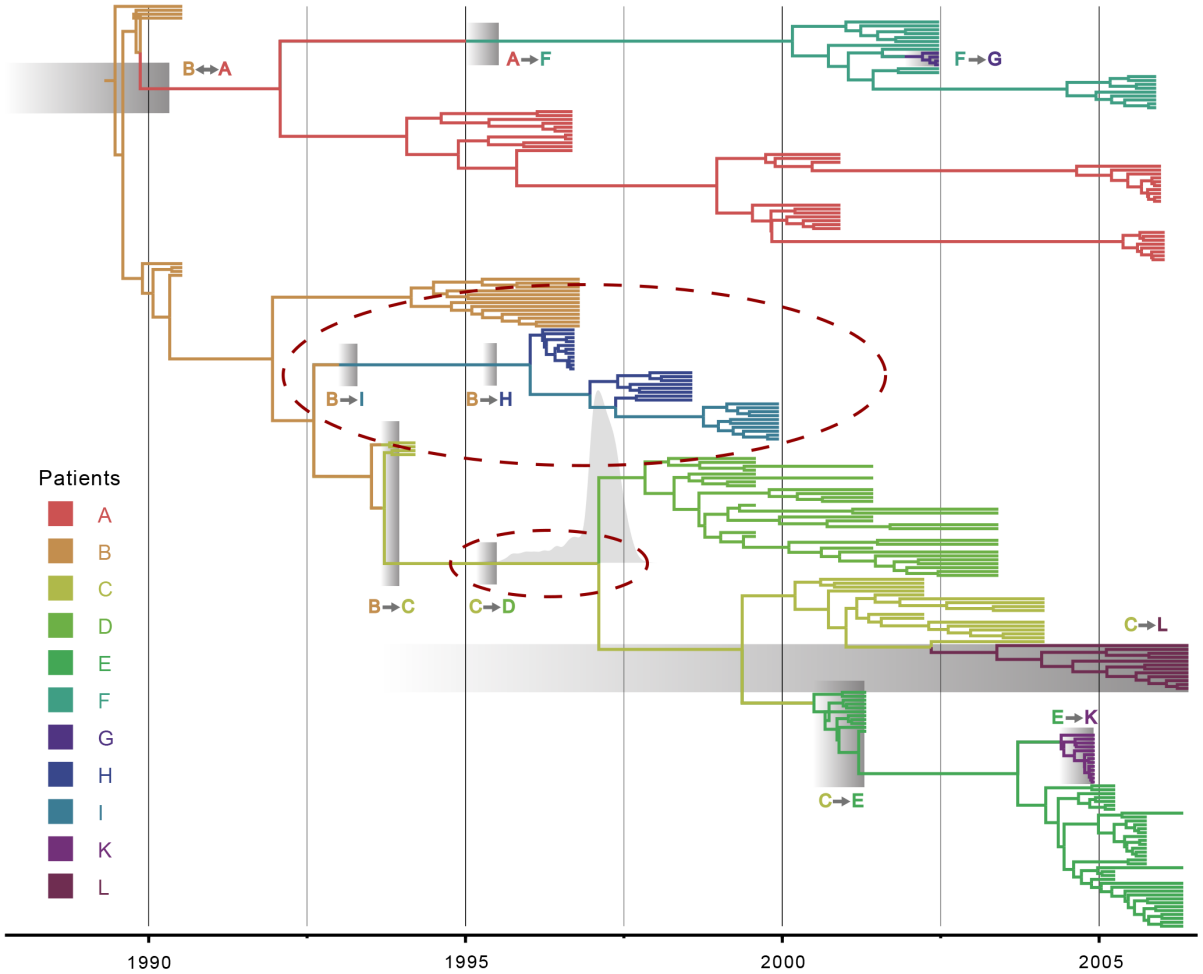
Maximum clade credibility tree for *pol*. Both tips and internal branches are colored according to the patient in which the viral lineages are hypothesized to reside (cfr. legend on the left). For branches that cross a transmission interval, the part up to the interval is assigned to the source patients. For posterior support values for each node, we refer to Figure S2. The transmission intervals are represented by the grey boxes. Dashed red circles indicate the topological and coalescent incongruences. For the C
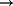
D coalescent event, the posterior marginal density of the tMRCA estimate is plotted over the corresponding node in greyshade. Only a lower limit of the transmission interval is known for the A
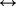
B transmission. For transmissions B
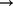
C, F
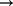
G, C
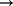
E, C
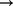
L and E
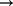
K the most recent boundary of the transmission interval (almost) equals the first sampling date.

Focusing on *pol*, we find maximum or close to maximum posterior compatibility probability for 7 out of 10 transmission events. To examine the incompatible coalescent patterns in more detail, we summarized a maximum clade credibility (MCC) tree and superimposed the transmission intervals in Figure 2. This reveals that an anomalous clustering pattern and not an inappropriate divergence time is responsible for the incompatibility for the patient B to H transmission event. The patient I cluster is nested within the patient H diversity, and following the transmission from patient B to patient I on the branch ancestral to I and H, the virus needs to remain in patient I up to its specific cluster. Therefore, the transmission from patient B to patient H, which would have to occur along the same branch as the B to I transmission, cannot be realized anymore.

Because the divergence time for patient B and patient I lineages is relatively close to the B
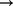
I transmission time, this coalescence event might occasionally be estimated after the transmission, resulting in a imperfect compatibility probability of 0.78. Although topologically consistent with the transmission history, the mean coalescence time for patient C and D lineages is estimated after the relevant transmission interval and only a small fraction of the posterior density for this estimate appears to be before the transmission time, resulting in a compatibility probability of 0.01.

**Figure 3:**
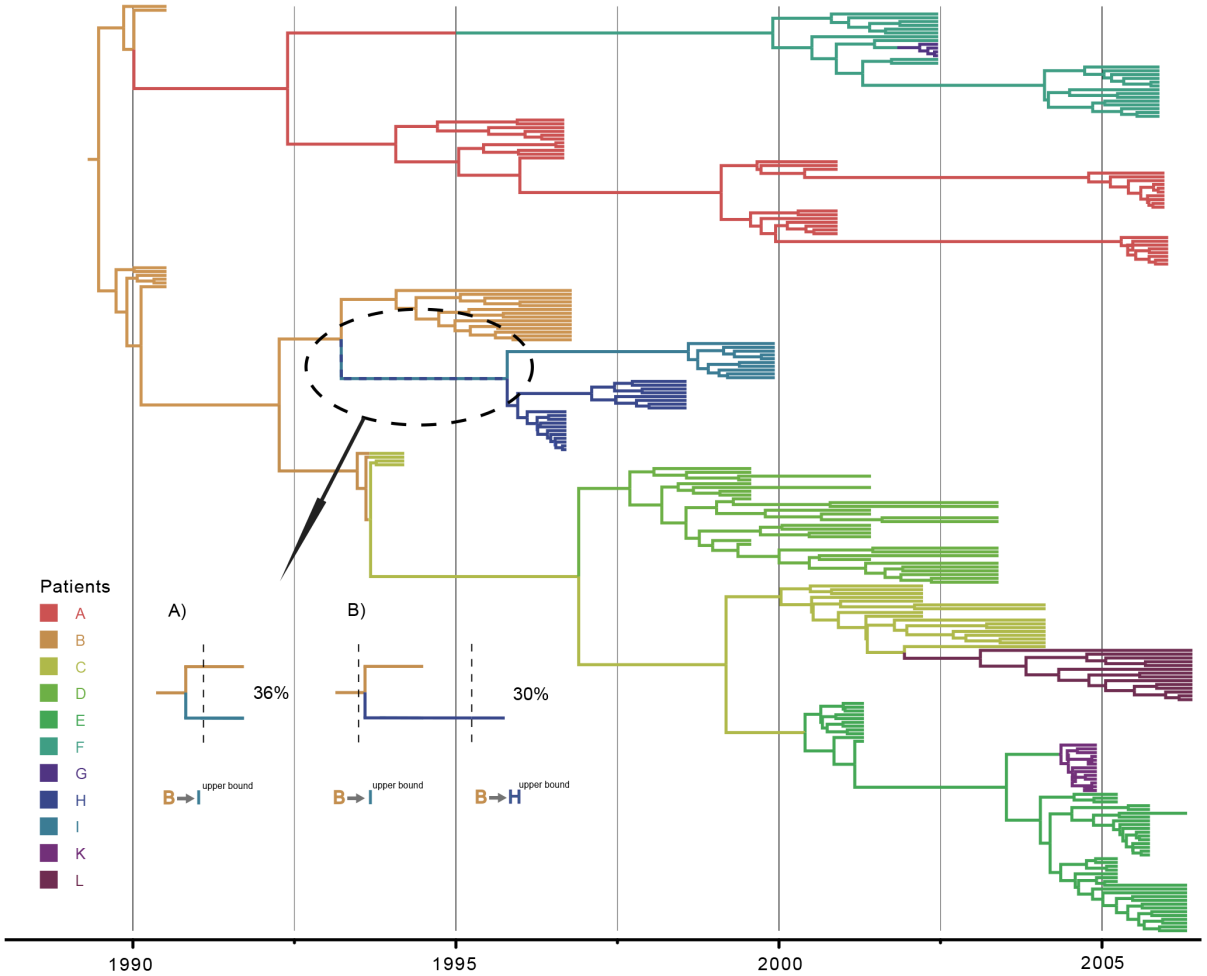
Maximum clade credibility tree for *pol* after exclusion of the same drug resistance associated positions as in [18]. Tips and internal branches are colored according to the states' posterior probability as estimated using a non-reversible discrete asymmetric trait analysis with the patients as discrete states [67], [68]. Both tips and internal branches are colored according to the patient in which the viral lineages are hypothesized to reside (cfr. legend on the left). For posterior support values for each node, we refer to Figure S3. The black dotted circle indicates the node responsible for the higher compatibility of the B
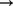
H transition. Like the increased C
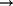
D compatibility, this results from the lower evolutionary rates that lead to somewhat older divergence time estimates. A) The divergence time between the patient B and I lineages is older than the upper bound of the B
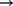
I transmission interval. Following the B
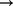
I host transition, B
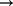
H cannot be compatible any more. B) When the B-I divergence time is estimated after its respective transmission, the viral genealogy is inferred to be incompatible with the B
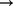
I transmission, and no transition into patient I is assumed. However, because the same node also represents the B-H coalescence time and this is in agreement with the compatibility constraints, the fair amount of too recent divergence time estimations for the B-I lineages results in 30% B
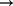
H compatibility.

To test whether drug selective pressure had any influence on compatibility, we performed the same analysis excluding positions associated with drug resistance [18]. Although this has little impact on most of the posterior compatibility estimates (Table 1), patient H and patient I viruses now form two reciprocally monophyletic clusters in the MCC tree and, as opposed to the unexpected paraphyletic clustering in Figure 2, this different clustering results in a somewhat higher compatibility for the transmission from patient B to H (see Figure 3). The evolutionary rate estimate is slightly lower than that for the original *pol* dataset (Table 1), which likely reflects the removal of positions at which substitutions can become rapidly fixed due to the drug selective pressure. As a consequence, some divergence times may also be slightly older, which may explain the somewhat higher compatibility probability for transmission from patient C to D, as well as for patient B to H (Figure 3).

**Figure 4:**
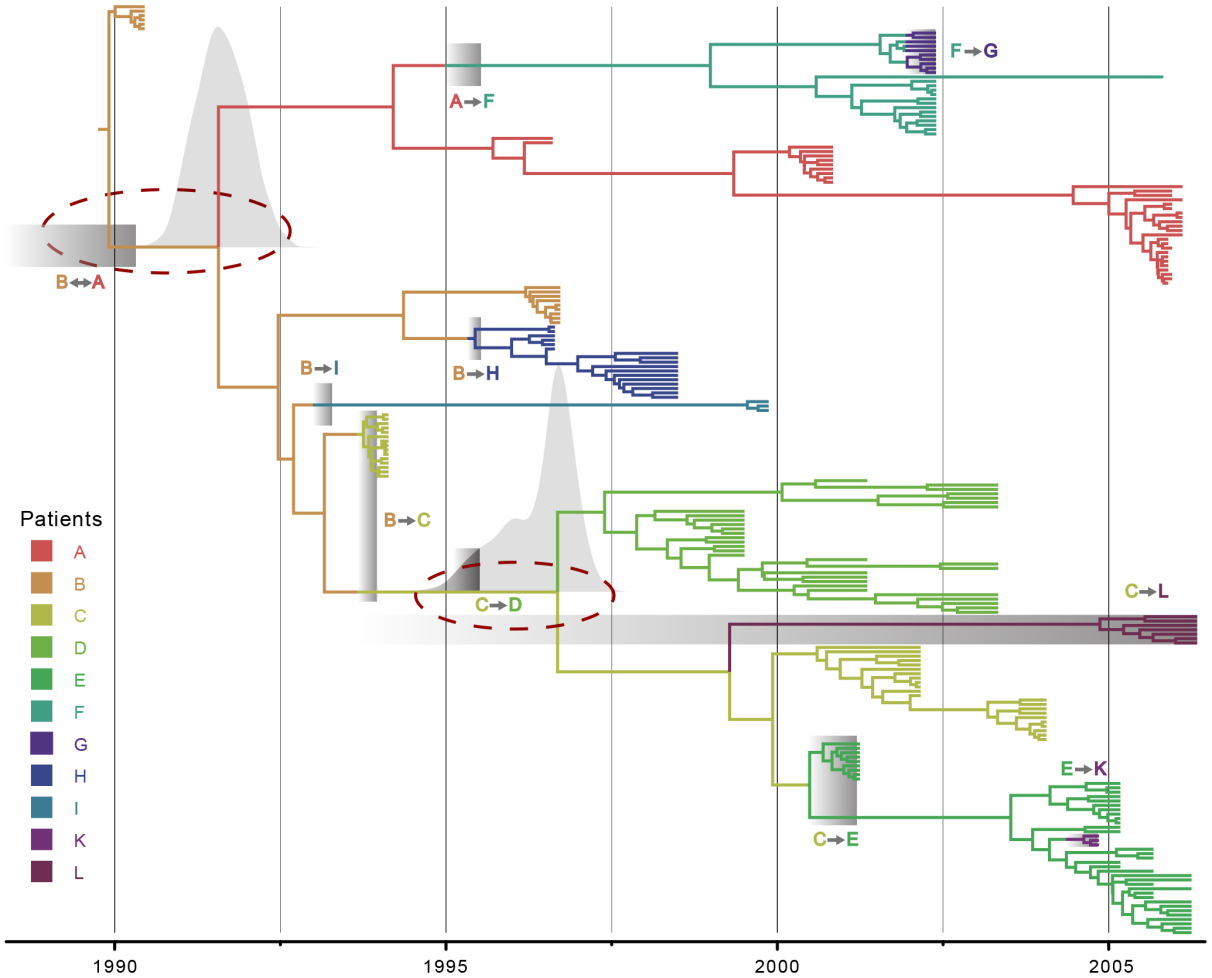
Maximum clade credibility tree for *env*. Both tips and internal branches are colored according to the patient in which the viral lineages are hypothesized to reside (cfr. legend on the left). For branches that cross a transmission interval, the part up to the interval is assigned to the source patients. For posterior support values for each node, we refer to Figure S4. The transmission intervals are represented by the grey boxes. Dashed red circles indicate the coalescent incongruences. For the A
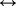
B and C
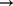
D coalescent event, the posterior marginal density of the tMRCA estimate is plotted over the corresponding node in greyshade. Only a lower limit of the transmission interval is known for the A
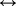
B transmission. For transmissions B
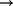
C, F
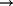
G, C
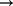
E, C
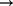
L and E
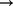
K the most recent boundary of the transmission interval equals the first sampling date.

Although the *env* compatibility is largely consistent with *pol*, notable exceptions exist. In particular, the B-H coalescent patterns are now fully compatible with the transmission history, and the *env* MCC tree (Figure 4) suggest that this results from a remarkable difference in clustering between patient B, H and I viruses. In this tree, both patient H and I viruses independently diverge from a patient B lineage and are not more closely related to each other anymore. The coalescent time for patient I and B lineages is now almost fully compatible with the known transmission time. For both the B
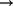
I and the original transmission event however, which either occurred from patient A to B or vice versa, there is now far less compatibility for either possible scenario, which results from a too recent MRCA for the relevant lineages relative to the transmission time (Figure 4). The compatibility probability for the C
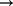
D transmission is also impacted by a too recent coalescent time of the relevant viral lineages, but to the same extent as for the *pol* analysis excluding drug resistance mutations.

#### Within-host dynamics, transmission bottleneck and transmission times under the transmission model

In addition to the compatibility constraints, the transmission model we develop as part of this study also specifies a within-host coalescent model with shared parameters across all patients, except maybe for the source (cfr. Methods). This can be implemented instead of a standard coalescent tree prior because the transmission times, which are estimable parameters constrained by the transmission interval boundaries, demarcate the patient-specific trajectories and their associated coalescent rates (cfr. Figure 1). We consider three simple parametric coalescent models: a constant population size model, an exponential growth and logistic growth model, with only the latter two accommodating a transmission bottleneck. To identify the most appropriate coalescent prior for the within-host population dynamics, we again use PS and SS estimates of the log marginal likelihood (Table S4). For both the *pol* and *env* data sets, a model with transmission-associated bottleneck consistently yields higher log marginal likelihoods than the constant population size model. The logistic growth model offers a better model fit than an exponential growth model in *pol* but this is not reproduced by the *env* analysis.

This model-fit evaluation supports a transmission bottleneck and suggests a relatively rapid increase in relative genetic diversity that levels off later in infection. The fact that we can capture signal for these dynamics is remarkable given the relatively sparse sampling of within-host diversity through time, in particular close to transmission, and the fact that treatment may impact genetic diversity. For these reasons, it is not surprising that it remains difficult to accurately estimate the overall transmission bottleneck in our transmission chain using the logistic growth model. Using a constant (between 0 and 1) 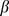
(1,1) prior for the ancestral proportion parameter, which quantifies the fraction of diversity transmitted from the source, we arrive at posterior estimates 0.32 [0.21–0.44] and 0.26 [0.00–0.70] for *pol* and *env* respectively. These estimates are unrealistically high compared to previous population genetic studies reporting on diversity loss at transmission [4] or single genome amplification studies demonstrating that HIV infections are generally initiated by a single or only a few variants [37]. Given the lack of clear bottleneck signal in our sampling, we can formalize previous knowledge as a prior distribution on the ancestral proportion and specify an increasingly higher prior probability towards small proportions using a 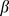
[1,10] and 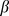
[1,100] distribution. As expected, the corresponding posterior estimates returned increasingly lower ancestral proportions (e.g. for *env* the mean decreases from 0.26 over 0.08 to 0.02 for 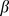
[1,1], 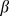
[1,10] and 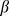
[1,100] respectively).

Because the transmission intervals we specify are generally rather narrow relative to the sampling density through time (e.g. for A
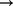
F, Figures 2 and 4), the transmission times are likely to be sampled uniformly from the known interval. However, occasionally we can find evidence that genealogical divergence times may also impact the transmission time estimates. For C
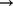
L for example, a very large time interval has been specified reflecting the uncertainty on the transmission time, in which case the lower boundary is determined by the divergence time between the source and recipient lineages. On the other hand, the estimates of divergence times relative to transmission times may also be affected by the bottleneck size. When considering the difference between the transmission time estimate and the time estimate for the MRCA of the recipient viral population for C
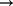
E in *env*, we obtain values of −0.05 (−0.68,0.24), 0.04 (−0.43,0.26) and 0.11 (0.00,0.30) years for the 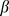
[1,1], 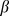
[1,10] and 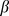
[1,100] priors respectively. The negative difference represents a MRCA of the recipient population that is older than the transmission time, and hence implies transmission of multiple variants, whereas the positive values represent transmission of a single lineage as expected by the prior specification preferring a transmission bottleneck. This reflects the potential interaction between bottleneck size and transmission/divergence times, but in the absence of strong prior specification, the extent to which this can occur will again depend on sampling intensity.

### Transmission decreases HIV-1 evolutionary rates

**Figure 5:**
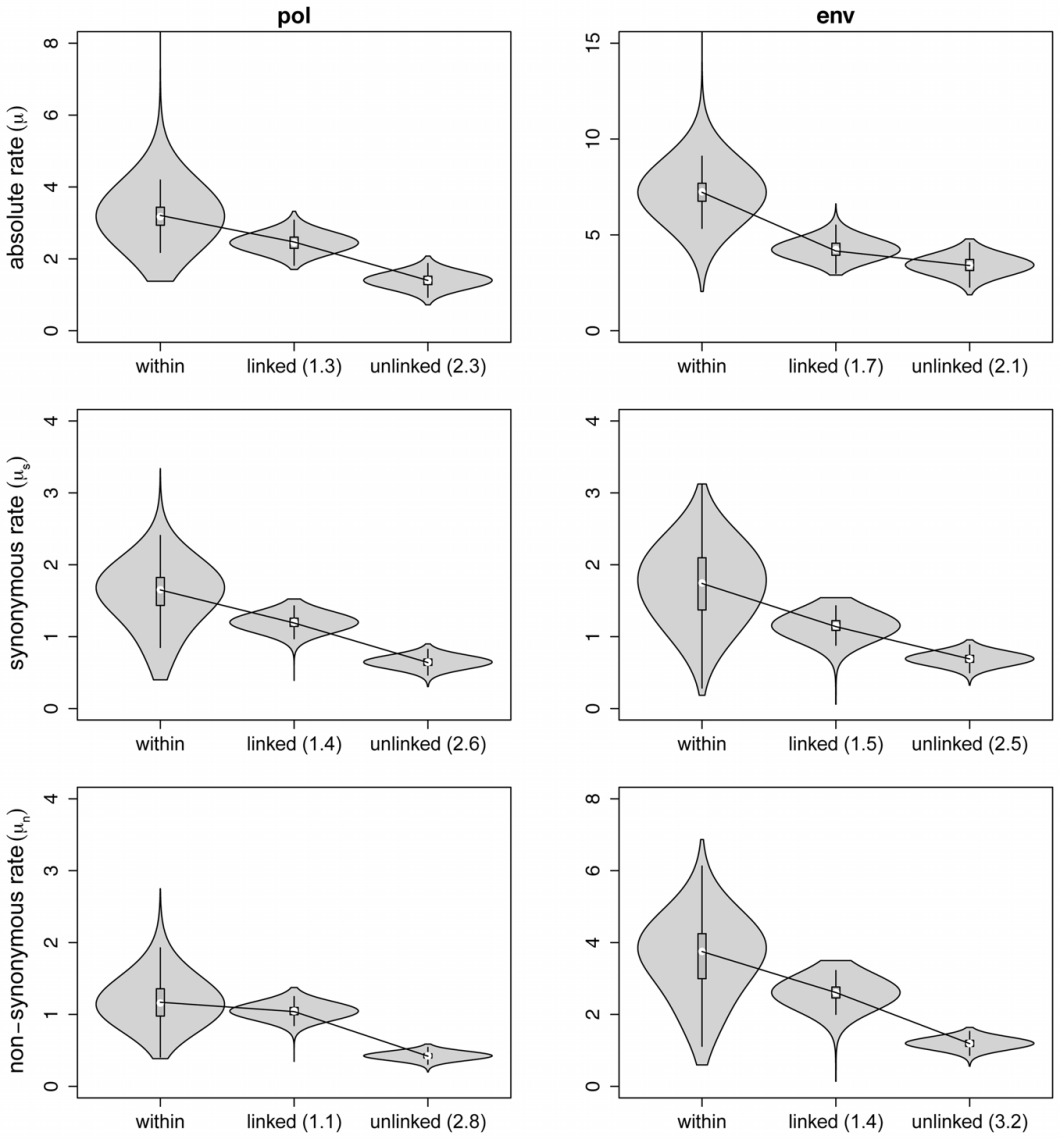
Violin plot representation of the *pol* and *env* within and between-host evolutionary rates. Only patients for which samples for more than one time point are available were used for the within-host analysis. The ‘within’ host label represents the HPM estimate of the mean within host evolutionary rate of all patients. Likewise, the ‘linked’ between host label marks the direct estimate of the between host evolutionary rate. For the latter, only the first available sample of each patient was used. The ‘unlinked’ between host labels denotes the rate estimates obtained with the skyride prior for the *pol* and *env* regions of the epidemiologically unlinked subtype C sequences that overlap with our clonal data sets. The means of each rate estimate are connected by a black line. Numbers between brackets indicate the fold decrease of the mean relative to the within host mean rate estimate. All rates are in units of nucleotide substitutions per site per year * 10^−3^.

Our sampling is not very informative about the transmission-associated bottleneck size, but it does provide a unique opportunity to investigate the impact of transmission on evolutionary rates. Although formal evaluations are sparse, evolutionary rates among hosts are suggested to be lower than evolutionary rates between hosts [27], [28], [30]. To investigate this using our data, we separately estimated within-host and between host evolutionary rates for both *pol* and *env*. The within-host estimate was obtained using a Bayesian hierarchical phylogenetic model (HPM) fit across patients for which multiple samples are available. The HPM model posits patient-specific evolutionary rate parameters, but allows sharing of evolutionary rate information across patients through a hierarchical prior specification. We report estimates of the mean of the population-level (hierarchical) distribution as the within-host evolutionary rate. An among-host evolutionary rate for closely related patients was obtained using a transmission model analysis that only considered a single sample per patient (cfr. Methods). In addition, we compared these inferences with evolutionary rate estimates from a data set representing epidemiologically-unrelated patients infected with subtype C for the same genome regions (cfr. Methods). Despite the uncertainty associated with evolutionary rate estimates, this reveals a clear rate decrease from small (within-host) to large (among-host) evolutionary scales (Figure 5), with an intermediate rate for the epidemiologically-related patients in the transmission chain. For both *pol* and *env*, we observe about a twofold decrease in evolutionary rate among epidemiologically-unrelated patients compared to the within-host evolutionary rate.

The patients in the subtype C transmission chain have received antiretroviral therapy for a substantial part of the period between the first and last sample (see Figure S1), and this can affect viral evolutionary rates in different ways [18], [38]. Because the rate estimate for the transmission chain relies on the samples before or only shortly after treatment initiation for each patient, we do not expect a considerable effect on the among-host evolutionary rate. To examine whether treatment biases the within-host evolutionary rates however, we compared our rates to estimates for a control set of longitudinally sampled therapy-naive patients (cfr Methods) using a Bayesian HPM approach with fixed-effects [39]. This does not support any rate differences (see Table S5), suggesting that therapy does not confound our comparison within and between hosts. Because the control data sets include sampling over different times during infection, we took the opportunity to also stratify these patients into an ‘early’ and ‘chronic’ group, based on sample availability before or after the first year of infection, in order to test for stage-specific evolutionary rates. No substantial rate difference between both groups was detected (see Table S5), which argues against evolutionary rate differences due to stage-specific selection.

To test more explicitly that transmission decreases evolutionary rates, we develop a new molecular clock approach that allows for rate variation according to a relaxed molecular clock model but also incorporates fixed effects to quantify a difference in rate along a specified subset of branches (cfr. Methods and Figure S5). We applied this model within the transmission chain framework in two different ways. First, we specified an estimable rate effect on the branches to which a transmission event can be unambiguously assigned and we estimate the support for a lower rate on these branches using ln Bayes factors (BFs). Using this approach, we find about a twofold lower rate on the branches that accommodate a transmission event in the subtype C transmission chain for both *pol* and *env* with a strong ln BF support (Table 2). We also extended the fixed effects to the complete branch set representing the transmitted lineage in the chain as opposed to the within-host branches that can be generally considered as evolutionary dead-ends (see Figure S5). This results in similar rate differences and associated ln BF support and complements our comparison of intra-host and inter-host evolutionary rates in providing statistical evidence for a slower among-host ‘trunk or backbone’ rate in the transmission chain compared to lineages that do not get transmitted. We note that this rate difference is not enforced by the transmission constraints because we get consistent results when using a flexible coalescent prior (the Bayesian skyride model, Table S6), even though *env* shows a somewhat less pronounced rate difference.

**Table 2:** Evolutionary rate estimates and support for the fixed effect in the mixed effects clock model.

Fixed effects	pol		env	
	rate	lnBF	rate	lnBF
transmission branches	1.82 (1.00,2.76)	7.50	3.95 (1.99,6.01)	6.40
within-host branches	4.28 (3.40,5.25)		8.41 (6.68,10.23)	
transmitted lineage branches	2.21 (1.57,2.99)	>7.50	3.80 (2.32,5.20)	>6.29
within-host branches	5.07 (4.02,6.21)		10.37 (8.06,12.76)	

The mean evolutionary rate and highest posterior density (HPD) intervals are expressed as the number of nucleotide substitutions (10^−3^) per site per year. The Bayes factor (BF) is computed as the posterior odds over the prior odds that the rate for the transmission branches or transmitted lineage branches is smaller than the within host-rate.

The different hypotheses that have been put forward to explain rate differences within and among hosts have different expectations concerning synonymous (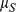) and non-synonymous (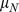) substitution rates [28]. Whereas ‘store and retrieve’ is expected to affect 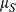 and 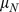 rates in a similar way, ‘adapt and revert’ and ‘stage-specific selection’ are predicted to have a greater influence on non-synonymous mutations and their substitution rates. Moreover, the latter two hypotheses may also imply a more pronounced rate decrease for non-synonymous substitutions in *env* because of the major immunological pressure it experiences. To assess these predictions for our data, we resort to recent techniques to map codon substitutions [40] and employ them to obtain posterior estimates of 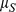 and 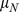 (see Methods). Comparing the 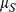 and 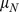 estimates for both *pol* and *env* to the overall substitution rates (Figure 5), we consistently find a similar rate decrease over the different evolutionary scales, and similar decreases for both *pol* and *env*. As expected for approximately silent substitutions, 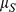 estimates are highly similar between *pol* and *env* for the same evolutionary scale (they are the only estimates with the same Y-axis scale for the corresponding *pol* and *env* panels in Figure 5). Estimates of 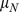 on the other hand are much higher for *env* due to stronger immune pressure and relaxed constraints in this gene region.

## Discussion

In this study, we present a novel transmission model in a Bayesian genealogical inference framework that focuses on time-calibrated viral evolutionary histories and requires such genealogies to be compatible with a known transmission history. Before applying the model to estimate HIV-1 evolutionary rates, we investigate the compatibility assumptions on new clonal data from a subtype C transmission chain and assess the model's potential to estimate transmission bottlenecks. We consider viral genealogies to be compatible with a transmission history if the viral lineages from the source and recipient coalesce before the time of transmission and if the host transitions can be superimposed onto the genealogy according to the time-ordered chain of transmission events. This approach follows gene-species tree thinking [19] and relaxes the assumption that viral and transmission trees need to be a perfect match, but explicitly incorporates temporal constraints instead.

The compatibility concept we introduce here, as well as the violations we identify in our data, are important considerations for phylogenetic studies that assess transmission linkage. Conditioning on the contact tracing information being correct, the major source for the 2 to 3 incompatible transmission events we observe for both *pol* and *env* appears to be a too recent divergence time estimate for the source-recipient lineages, as exemplified by the C–D coalescence patterns, and not anomalous clustering. In this respect, it is important to note that high compatibility statistics are only expected if a considerable ancestral divergence (or pre-transmission interval [41]) exists for each transmission event. If the source and recipient lineages coalesce almost immediately before the time of transmission, the stochasticity of the substitution process and the stochastic error in the divergence time estimates will inevitably result in credible intervals for the divergence time of source-recipient lineages that overlap with the upper boundary for the transmission time. However, since the ancestral divergence is generally pronounced in transmission chains [41], the fact that we do not observe high compatibility for these transmission events may be due to the same reason we invoke for the lower inter-host evolutionary rates. A preferential transmission of ancestral viruses may in fact result in more similar source-recipient lineages than expected based on their transmission time and bias their divergence time estimates towards more recent times (see Figure S6). The only instance of incompatibility that appears to result from a clustering issue involves the clustering of patient B,H and I lineages in *pol*. The marked difference with the clustering for *env* might have resulted from a pattern of convergent evolution leading to higher similarity between patient I and patient H virus in *pol*. An analysis excluding the positions associated with drug resistance indicated that drug selective pressure may at least have been responsible for the unexpected paraphyletic clustering of patient I with respect to patient H, but their divergence time is still too recent to be compatible with patient B as a source for both these patients. We note that convergent evolution due to drug selective pressure also induced incompatible clustering in the original analysis of the population sequences from this transmission chain [18]. However, this concerned the viruses from patients F and G, and the convergent substitution patterns involved may have a lower impact on our analyses because we use longer and therefore more informative clonal sequences. Although we attempted to exclude recombinant sequences from our analysis, we note that undetected recombination within a gene region may also be responsible for incompatibly between the viral genealogy and transmission history.

Also relevant to phylogenetic investigations of viral transmission is the ability to infer transmission direction. A recent study of HIV transmission in two criminal cases suggested that transmission direction can be deduced from paraphyletic relationships that show recipient virus clades nested within the larger diversity of the source virus population [20]. We demonstrate that such relationships can be easily reconstructed in a rooted phylogeny when source samples are available before and after transmission. In the absence of such samples from the source, however, both the source and recipient diversity may need to be sampled close to transmission to be able to recover paraphyletic relationships. We show that different gene regions are not necessarily consistent in revealing recipient sub-clusters within a source clade. For example, a paraphyletic relationship is reconstructed for the F
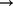
G transmission in *pol* but not in *env*. In source F, a selective sweep in the *env* region, which is the dominant target for immune selective pressure, might already have erased the paraphyletic structure. Indeed, within-host HIV phylogenies generally have a strong temporal structure [42] and the continual strain turnover will reduce the probability of recovering source-recipient paraphyletic relationships. In addition to the differential impact of selective pressure (e.g. drug selective pressure in *pol* and immune selective pressure in *env*) and incomplete lineage sorting effects in the two genome regions that may be largely unlinked due to recombination, also experimental aspects leading to non-proportional representation of variants could explain the general differences we observe among the two gene regions.

The transmission model incorporates a coalescent prior that models the within-host population dynamics for each patient starting from transmission from its respective source. Although not the focus of our study, we demonstrate that a model incorporating a transmission bottleneck with subsequent logistic growth in relative genetic diversity fits our data best. Accurately quantifying the bottleneck, however, remains challenging and requires more dense sampling, in particular close to transmission. In the absence of such data, prior information on the bottleneck size may be incorporated, which in our case indicates that the bottleneck size parameter may interact with the relative timing of the transmission and recipient MRCA, provided recipient diversity is sampled close to transmission. The latter is likely to be a general requirement to accurately estimate HIV-1 infection dates from recipient coalescent times [22]. Intensive sampling throughout transmission will not only assist in estimating transmission times or quantifying bottlenecks, but it may also help to resolve whether the bottleneck results from a single variant being transmitted as opposed to the outgrowth of a single lineage from multiple transmitted viruses in the recipient [43]. In addition to more samples, the genealogical inference may also benefit from a more detailed characterization of the diversity within each sample. Nowadays this can be efficiently pursued using next-generation sequencing (NGS) platforms, although this would result in shorter read lengths than the clonal sequences we obtained here. Finally, conventional (RT-)PCR followed by either molecular cloning or NGS may both suffer from a non-proportional representation of sequences due to the re-sampling of only certain templates. This as well as other confounders can be avoided by using single genome amplification followed by direct sequencing of the amplicons [44]. We note that the availability of more comprehensive sampling may not only better inform the current model but also stimulate the development of extensions such as patient-specific coalescent parameterizations as well as more complex coalescent models, perhaps in a hierarchical framework [39].

Using the transmission model, we scrutinize HIV-1 evolutionary rates in the subtype C transmission chain and find an intermediate rate compared to within-host evolution on the one hand and evolution among epidemiologically unrelated individuals on the other hand. This suggest that the more transmissions in the HIV-1 evolutionary history, the slower the evolutionary rate, which may be consistent with the different hypotheses put forward to explain a rate mismatch at different HIV-1 evolutionary scales. The subtype C transmission chain encompasses 15 years of HIV evolution and 10 transmission events, while the subtype C evolutionary history for 81 sequences from unrelated patients encompasses about 50 years of HIV evolution but at the very least more than 8 times the number of transmission events. The fact there are far more transmission events and therefore more opportunity for transmission-associated rate decrease in the latter explains why we find the lowest evolutionary at this scale. We test the transmission-associated rate decline more explicitly by applying a new molecular clock model that allows quantifying a different rate for the branches that accommodate a transmission event. In agreement with the twofold lower rate among epidemiologically-unrelated patients compared to within-host evolutionary rates, we demonstrate a similar rate difference between branches accommodating a transmission event or branches representing the entire transmitted lineage compared to background within-host evolution in the viral genealogy for both *pol* and *env*. This suggests that lineages that avoid accumulating particular substitutions within hosts, perhaps those resulting from the evolutionary arms race, do not compromise their transmissibility and will consequently be characterized by a lower divergence rate.

Evidence for a rate difference within and among hosts across the entire genome was recently interpreted as support for the ‘store and retrieve’ hypothesis [30]. Indeed, it seems unlikely that the selection forces invoked by both the ‘stage-specific selection’ hypothesis and the ‘adapt and revert’ hypothesis operate strongly across the entire genome. Our study also finds similar differences in two different genome regions, but more importantly, we provide evidence for a similar decline in both synonymous and non-synonymous substitution rates. This argues more directly against hypotheses based on selective dynamics whereas it is compatibility with the ‘store and retrieve’ hypothesis. We acknowledge that synonymous substitutions are not necessarily selectively neutral, for example due to codon usage bias and secondary RNA structure, but the selection effect will still be considerably weaker on silent versus replacement changes [45]. It is therefore not surprising that we find similar synonymous substitution rates for both *pol* and *env* at the same evolutionary scale despite very different non-synonymous rates. By focusing on synonymous substitutions, we also avoid having to compare rates for a subset of branches, such as the internal branches [30], which, unlike external or tip branches, are less likely to represent transient (slightly) deleterious mutations that will be eliminated by purifying selection [46], [47]. Whereas [30] found a more pronounced rate difference in the *env* gene, suggesting that reversions may also contribute to the rate difference in this gene, we find similar rate differences for both *pol* and *env*. However, our study focuses on the gp41 region of the *env* gene which may experience less reversions compared to the C2V5 region of *env* gp120 for example.

By focusing on subtype C, our study extends the rate differences within and among hosts that were previously established for subtype B. However, the rate mismatch between the intra-host and interhost level for epidemiologically unrelated patients appears to be less pronounced (about 2-fold) than that identified for subtype B complete genomes (about 4 to 5 fold difference, [30]). This discrepancy may be due to the differences in the transmission dynamics underlying subtype C and subtype B spread. Based on a recent study that provided evidence against preferential transmission from the compartmentalized virus [48], and on rates of evolution that are even slower among IDUs than among populations where the virus is transmitted sexually [31], Lythgoe et al. [28] claim that an inherent transmission and/or establishment advantage is the most plausible hypothesis and speculate that larger inoculum sizes during high-dose rectal and intravenous transmission may result in slower among-host rates than for sexual transmission. In the latter case, stochastic effects may be more important. Following this argumentation, the less pronounced rate mismatch we find for subtype C may be due to the largely heterosexual nature of the this epidemic as opposed to a larger contribution of homosexual and intravenous drug user (IDU) transmission for subtype B. However, we note that a comparison of six subtype B within-host data sets for the *pol* region also pointed at lower differences (1.64 fold; [30]).

While the role of latently-infected memory T cells in creating a long-term viral reservoir was already well established as a significant barrier to HIV eradication [49], the ‘store and retrieve’ hypothesis also attributes a major role to HIV persistence and reservoir dynamics in the conflict between HIV selective pressures at the within and between host level. HIV evolution and adaptation within a particular host has been termed ‘shortsighted’ because it is unlikely to favor viral variants that are efficiently transmitted or that efficiently establish infection in new hosts [50]. The storage of HIV variants in latent cells at an early stage and preferential transmission upon reactivation later in infection provides a mechanism to respond to the different selective pressures within and between hosts [51]. Further studies need to determine how pervasive ‘store and retrieve’ can be because it has important implications for modeling the spread of drug resistant and immune escape variants. Our analysis of an HIV transmission cluster using dedicated Bayesian inference approaches corroborates recent findings about rate differences within and among hosts and hints at potential differences between different subtypes, perhaps linked to differences in main risk group-associated transmission routes.

## Materials and Methods

### Amplification, cloning and sequencing of the partial *pol* and *env* gene regions from the HIV-1 subtype C transmission chain samples

We obtained PCR products for both the *pol* and *env* gp41 region (HXB2 nucleotide positions 2097 to 2292 and 7173 to 8792 respectively) using previously described procedures that were specifically adapted for the use of Expand High Fidelity PCR System (Roche Diagnostics, Mannheim, Germany) [52], [53]. PCR products from 25 samples were cloned using TOPO XL PCR cloning kit (Life Technologies, Gent, Belgium), and 1–19 clones were subsequently sequenced. TOPO ligated PCR fragments were transformed into TOPO 10 cells (Life Technologies, Gent, Belgium). Single colonies were used to inoculate 5 mL LB aliquots and left overnight in a shaking incubator at 37°C. Plasmid DNA was extracted from cultured cells using a QIAprep Miniprep Kit (Qiagen, Venlo, The Netherlands) and clones were sequenced using an ABI PRISM Big Dye Terminator v3.1 Ready Reaction Cycle Sequencing Kit with previously described primer sets [52], [53]. Sequencing reactions were run on an ABI3100 Genetic Analyzer (Life Technologies). Sequence fragments were assembled and analyzed using Sequence Analysis v3.7 and SeqScape v2.0 (Life Technologies, Gent). For *env* in particular, the clone sequences were considerably longer than the previously obtained population sequences [18] because numerous insertions and deletions seriously hamper unambiguous population sequencing [53]. Testing both datasets for recombination signal with the 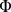-test [54] using SplitsTree v.4.12.6 [55] revealed significant recombination signal (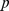 = 8.328E-5 for *env* and 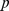 = 6.248E-4 for *pol*. We omit sequences with statistically significant recombination signal, as identified using RDP3 [56], from further analyses.

### Bayesian evolutionary reconstruction of the HIV-1 subtype C transmission chain

Sequences were aligned using Clustal W [57] and manually edited according to their codon reading frame in Se-Al (http://tree.bio.ed.ac.uk). Because identical clones might have resulted from template re-sampling [58], especially at lower viral loads, we analyzed only unique sequences obtained from the isolates. We conducted Bayesian evolutionary reconstructions using BEAST for both the *pol* and *env* gp41 alignments employing either the Skyride model [33] or the transmission model discussed below. The Skyride model was used as a flexible demographic tree prior in analyses aimed at testing molecular clocks and evaluating the coalescent compatibility of viral genealogy with the known transmission history, before enforcing this compatibility in analysis using the transmission model. The latter is based on the compatibility concept outlined below as the first part of the transmission model, and formalized into a compatibility statistic. Specifically, we record a statistic for each transmission in each sampled genealogy that evaluates whether the source-recipient coalescent events pre-date the specific transmission event and whether the correct host transition order can be superimposed onto the viral genealogy, allowing us to calculate the posterior compatibility probability for each transmission event. We perform our analyses with a codon position partitioning into first+second and third positions, each associated with a general-time reversible (GTR) model and among site rate heterogeneity modeled using a discrete 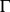-distribution and a proportion of invariable sites. We apply the same substitution model and among-site rate variability to the data discussed in the next sections. MCMC chains were run sufficiently long to ensure convergence, as inspected using Tracer v1.5 (http://tree.bio.ed.ac.uk). Maximum clade credibility (MCC) trees were summarized using the TreeAnnotator tool in BEAST and visualized in FigTree v1.4 (http://tree.bio.ed.ac.uk). Molecular clock models, including a strict clock assumption as well as the uncorrelated relaxed clock models with underlying exponential (uced) and lognormal distribution (ucld), were tested using recent implementations of path sampling (PS) [59] and stepping-stone (SS) sampling [60] estimators of the marginal likelihood in BEAST [35]. Both PS and SS have been shown to outperform the widely-used harmonic mean estimators [35] and offer similar performance as Bayesian model averaging when proper priors are used [36]. The length of each power posterior MCMC length in the PS/SS approach was set to he number of states for the standard MCMC analysis divided by the number of steps taken to arrive at the prior.

### A Bayesian evolutionary model for known HIV-1 transmission histories

We implement a new genealogical model in the BEAST statistical inference software [61] that accounts for the known transmission links among patients and allows estimating evolutionary parameters as well as transmission times and within-host population dynamics from viral diversity sampled through time. BEAST infers rooted, time-calibrated genealogies with a coalescent or birth-death process as a prior distribution for the branching events [61]. Generally, the entire genealogy is assumed to be generated by a single coalescent or branching process. To accommodate the specific transmission structure and within-host population dynamics, we modify this standard prior specification in two ways. First, we enforce the viral genealogy to be compatible with the known transmission history by enforcing coalescent events between source and recipient lineages to exist before the transmission time and assuming a transmission-associated source-recipient host transition along the relevant lineages in the viral genealogy. In Figure 1, we illustrate the coalescent compatibility concept for a hypothetical transmission chain of 4 patients and a particular genealogy of viruses sampled from each patient. For each transmission event, we show an upper and lower boundary for the transmission event which represents the fact that the actual transmission time is difficult to pinpoint, but a transmission interval can often be defined using external information (e.g. based on the last negative and first HIV positive test for the recipient). In this case, we require the coalescent times for source and recipient lineages to predate the upper boundary for the transmission time and superimpose transmission-associated host transitions onto the viral genealogy as depicted by the transitions in branch colors in Figure 1. The latter allows tracking the host transition history in the viral genealogy and ensures that also inadequate clustering of patient-specific lineages can lead to incompatibility despite the fact the relevant coalescent times may still be compatible with their transmission time (as observed for the patient H and I lineages in the *pol* genealogy in the subtype C transmission cluster). In the example genealogy, both the coalescent events for lineages from patient 1 and patient 2, and from patient 2 and patient 3, are compatible with their transmission events, despite the fact that two lineages are transmitted during the latter event. In contrast, the most recent common ancestor for lineages from patient 3 and patient 4 appears to be more recent than the upper boundary for the transmission time between patient 3 and patient 4, which is considered to be incompatible under our model. Therefore, no genealogies would be allowed to have such coalescent patterns under the transmission constraints.

We explicitly parameterize the transmission times for each transmission event and integrate out their dates over the known transmission time intervals in our inference framework. The transmission time parameters naturally partition the genealogy into patient-specific lineages represented by the different branch colors in Figure 1. This allows us to model a within-host coalescent process for each patient and estimate population parameters based on the distribution of waiting times in the patient-specific lineages. Because within-host sampling is generally sparse for transmission clusters, all patients share the same coalescent model in the current implementation of the model, except maybe for the ultimate source of the transmission cluster which cannot be related to its respective source patient. We consider simple demographic functions as coalescent models, including constant population size:
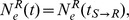
where 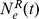 is the effective population size in the recipient (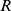) at time 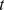 and 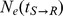 is the effective population size at time of transmission (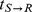) from the source (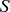) to 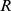. Because this model assumes no transmission bottleneck (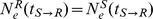), where 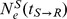 is the effective population size in 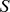 at 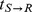), we also consider an exponential growth model:
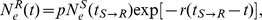
where 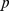 represents an ancestral proportion (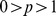) of the effective population size in 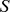 at time of transmission and 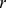 represents the exponential growth rate, and extend this further to a logistic growth model:
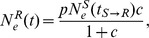
with
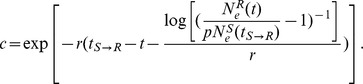



So, the latter two demographic functions are explicitly parameterized in terms of a transmission bottleneck. Because this cannot be applied to the patient at the origin of the transmission chain (e.g. patient 1 in Figure 1), we allow specifying a separate demographic function for this patient using standard parametric formulations. Although constant, exponential and logistic models can therefore also be applied to this patient, we consistently opted for a simple constant model because the putative source patients are only sparsely sampled through time (see Figure S1). Our BEAST implementation enables the simultaneous inference of viral genealogical history, including the tempo and mode of viral evolution, and transmission times and bottlenecks. We sample from the posterior distribution using Markov chain Monte Carlo (MCMC) incorporating standard transition kernels.

### Comparing estimates of within and among host evolutionary rates for the subtype C transmission cluster data

In order to compare evolutionary rate estimates at different scales, we distinguish between HIV-1 evolution within hosts, among epidemiologically-related hosts and among epidemiologically unrelated hosts. We study the first two processes based on data from the subtype C transmission cluster and discuss the data and associated analysis for the remaining evolutionary scale in the next section. To obtain a ‘pure’ within-host evolutionary rate estimate for *pol* and *env* gp41 across different patients, we apply a Bayesian Hierarchical Phylogenetic (HPM) model to the transmission cluster patients for which sequences from multiple time points are available (see Table S2) [62], [63]. This approach allows specifying independent genealogies for each patient while pooling information on evolutionary and population genetic parameters across patients through hierarchical prior specification. Due to the sparse sampling within the patients, we resort to a strict molecular clock model and apply a constant population size model to the patient-specific genealogy. We specify hierarchical prior distributions over the evolutionary rate and demographic parameters, allowing them to vary around an unknown common mean. We consider the mean estimate of the hierarchical prior distribution for the evolutionary rate as a quantification of the overall within-host evolutionary rate. To reduce the impact of within-host evolution among the epidemiologically-related patients, we only include the time point of each patient closest to the transmission event from its source.

Because HIV-1 replication rate and evolutionary rate may be affected by drug treatment, which was common for the patients in our subtype C transmission chain, we sought to investigate how comparable our within-host rate was to estimates from untreated patients. For this purpose, we compiled control data sets based on a search for intra-patient sequences in the HIV sequence database (http://www.hiv.lanl.gov/) according to the following criteria: (i) longitudinal samples, (ii) known time of sampling, (iii) untreated, (iv) *pol* or *env* genome region, including fragments with a minimum length of 200. By screening the relevant publications, we identified 10 and 15 studies with *pol* and *env* data respectively that met our criteria. To ensure a close match in genomic region, all sequence data were aligned against the clonal data. Only sequences with >75% overlap with the clonal data of the respective region were kept for further analysis. Because only few *pol* sequences (9/30 patients) spanned the entire length of the clonal sequence alignment, we trimmed the alignment from 421 AA to 330 AA ( = 78.5% of the original length). Finally, duplicates were removed and sequences were grouped per patient. This resulted in the inclusion of 30 patients for which the serially-sampled sequences start at AA 1 of protease and end at AA 231 of reverse transcriptase (numbering according to HXB2). For *env*, where the sequence overlap was less of an issue, the intermediate alignment consisted of 34 patients and comprised AA 468 to AA 856 of gp160. We refer to Tables S7 and S8 for a detailed overview of all control datasets.

In addition to serving as untreated controls, both the *pol* and *env* data sets were stratified in those sampled during ‘early’ and ‘chronic’ stage of infection by making use of the available disease stage information, in order to assess what impact this has on substitution rates estimates. In particular we classified sequences under ‘early’ when they were sampled in the first year of infection and ‘chronic’ when they were sampled later in infection. The data from the subtype C transmission chain corresponds better with data from the chronic stage of infection, which generally also allows for sampling over longer time periods providing potentially more calibration information. Following the within-host evolutionary analysis for transmission chain patients, we apply a Bayesian HPM procedure to this data, but extend it with fixed effects to test for differences among patient groups [63]. The fixed-effects HPM enables the estimation of Bayes Factor (BF) support for the early vs. chronic group effect on any evolutionary parameter, in our case the evolutionary rate.

### Evolutionary rate estimation for epidemiologically-unlinked subtype C sequence data

To complement our rate estimates within hosts and among epidemiologically-linked hosts, we compile a representative subtype C data set from epidemiologically-unlinked hosts. To this purpose, we retrieve and align all available HIV-1 subtype C full genomes with annotated sampling year from the Los Alamos HIV sequence database (http://www.hiv.lanl.gov/). From the resulting alignment containing 505 sequences, we select a diverse subset to minimize epidemiological relatedness and ensure that they are representative for the diversity of the subtype C epidemic. At the same time, we aim to spread the sampling density over the available sampling time interval. Therefore, we select the 5 most divergent sequences within each sampling year by constructing a BioNJ tree in Seaview [64], followed by subsampling according to diversity. For the latter we make use of the Phylogenetic Diversity algorithm, which selects for the subtree of 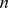 taxa connected by the longest branch length [65]. For the year 1989, we kept only two sequences in our selection because 3 of the 4 available sequences were from the same patient. This selection procedure resulted in a dataset of 82 taxa spanning the period of 1986–2010. Inspection of the temporal signal by plotting root-to-tip divergence as a function of sampling time in Path-O-Gen v1.3 (http://tree.bio.ed.ac.uk/) lead us to remove 1 outlier sequence from 2009, and showed clear signal for divergence accumulation over the sampling time interval (R^2^ = 0.50) for the remaining 81 full genomes. We again used the 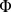-test [54] as implemented in SplitsTree v4.12.6 [55] to detect recombination; no significant signal was found.

For the Bayesian genealogical inference, we partition the full genome by gene to allow for among-gene rate variation. We further subdivide the *rev* and *tat* genes according to their splicing parts, and split *pol* and *env* into the region that overlaps with our clonal data and the remainder of the gene. We specify a Bayesian HPM for the gene-specific GTR substitution model parameters, the shape parameter for 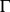-distribution modeling among-site rate variation, and for the proportion of invariant sites. Similar to the analyses of the other data sets, we specify a Bayesian Skyride model as a flexible demographic prior for the tree.

### A mixed effects molecular clock to model among-lineage evolutionary rate variation

To quantify and test for different evolutionary rates along an arbitrary branch set in the genealogy, we develop a novel mixed effects molecular clock approach in our Bayesian framework that combines both fixed and random effects. Following standard hierarchical modeling terminology, the random effects 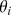 quantify possibly different rates for each branch 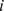 and we posit that these effects arise from an uncorrelated relaxed clock process following [34] on the log-scale. Assuming effects are additive on the log-scale, we further incorporate fixed effects 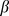 to allow for different overall rates on fixed subset of branches in the unknown genealogy. Specifically, we model the overall rate 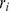 on branch 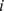 as
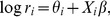
(1)where 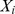 is the fixed design indicator or covariate associated with branch 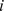.

We test two different fixed-effect designs for the transmission chain data: one that differentiates branches along which a transmission event occurred from the remaining branches and one that differentiates the branches representing the transmitted lineage from the remaining branches (see Figure S5). In the former case, we focus on single branches that unequivocally represent a transmission event. For these branches, we set 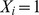; for all other branches, we set 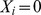. To achieve unequivocal events, we omit the sequence samples from patient G and the earliest sampling time point for patient C (C94), which complicate the unambiguous assignment of transmission events. The remaining nine transmission-associated branches are generally well-supported in the posterior according to the genealogical inference, but we enforce the descendent taxa to be monophyletic in the molecular clock inference to ensure that the effect is always associated with an identifiable branch. For the second approach, we specify ‘transmitted’ lineages for the full data set without monophyletic constraints as the set of branches from the root of the tree to the MRCAs of patients from which there is no more onwards transmission in the chain; these branches receive 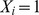 and represent the ‘trunk’ or ‘backbone’ lineages [47] for multiple patients in a transmission chain.

To evaluate the significance of the fixed-effect specification, we conduct a Bayes factor (BF) test [66] that expresses the posterior odds over the prior odds that rates on the branches of interest (transmission-associated or transmission lineage-associated) are lower than the background within-host branches. We perform the BF test using the posterior sample obtained via MCMC directly since the restricted hypothesis is nested within the unconstrained model that we simulate. Under the unconstrained model, the posterior sample average of the indicator 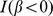 converges to the posterior probability of the constrained hypothesis. Since the prior odds in our case simplify to 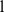, we simply need to compute the odds ratio of the mean indicator value to estimate the BF.

### Estimating absolute rates of synonymous and non-synonymous substitutions at the different evolutionary scales

To estimate absolute synonymous and non-synonymous substitution rates, we integrate recently developed stochastic mapping procedures in the BEAST analyses described above [40]. We follow an approach that is conceptually similar to [47], but is computationally more efficient in accommodating the uncertainty about the phylogenetic tree and about other nuisance parameters. Briefly, we fit codon position partitioned substitution models in a Bayesian framework and use standard MCMC integration to obtain a sample from the posterior distribution of model parameters. At each iteration of the MCMC, we use stochastic mapping to impute the full evolutionary history of each nucleotide position within each codon site in our alignment and subsequently summarize the resulting numbers synonymous (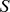) and non-synonymous (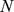) substitutions. To obtain posterior estimates of synonymous (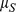) and non-synonymous (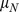) evolutionary rates in substitutions per site per year, we divide the total 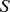 and 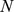 counts at each iteration by the total tree length in time units, and summarize these quantities across the posterior distribution of trees to arrive at mean estimates and credible intervals. To obtain an overall within-host 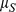 and 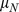 estimate for comparison with the estimates for the epidemiologically-linked and epidemiologically-unrelated data sets, we sum the 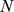 and 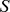 counts for the patient-specific genealogies at each iteration in the HPM analysis and divide them by the sum of the respective tree lengths, and then also summarize these quantities across all samples.

## Supporting Information

Figure S1 **Schematic representation of the studied transmission chain.** Orange arrows indicate transmission events. The width of the arrow is proportional to the time interval for transmission. For the first event between patient A and B, the time and direction of transmission could not be established by the patient interviews nor by clinical data. This is indicated by the double-sided arrow. The patients are indicated by bars whereby the color indicates the treatment status. Red indicates periods without treatment whereas the treatment type is as in the legend. †: the patient is deceased. The light blue arrows indicate sampling events for which clonal data were generated, including the sampling year.
(EPS)

Figure S2 **Maximum clade credibility tree for** ***pol*** **.** Tips and internal branches are colored according to the states posterior probability as estimated using a non-reversible discrete asymmetric trait analysis with the patients as discrete states [67], [68]. This was run on the empirical tree distribution obtained with the Skyride analysis of our chain data. The correspondence between the colors and patients is as in the legend. Numbers indicate the posterior probability of the nodes.
(EPS)

Figure S3 **Maximum clade credibility tree for** ***pol*** **after exclusion of the same drug resistance associated positions as in** [**18**] **.** Tips and internal branches are colored according to the states posterior probability as estimated using a non-reversible discrete asymmetric trait analysis with the patients as discrete states [67], [68]. This was run on the empirical tree distribution obtained with the Skyride analysis of our chain data. The correspondence between the colors and patients is as in the legend. Numbers indicate the posterior probability of the nodes.
(EPS)

Figure S4 **Maximum clade credibility tree for** ***env*** **.** Tips and internal branches are colored according to the states posterior probability as estimated using a non-reversible discrete asymmetric trait analysis with the patients as discrete states [67], [68]. This was run on the empirical tree distribution obtained with the Skyride analysis of our chain data. The correspondence between the colors and patients is as in the legend. Numbers indicate the posterior probability of the nodes.
(EPS)

Figure S5 **Illustration of the fixed-effects rate specification using** ***env*** **as an example.** Branches on which the rate effect is specified are coloured red. A) The rate effect is specified on the branches over which transmission could unambiguously be assigned. B) The fixed-effects are specified on the branches that represent the transmitted lineages.
(EPS)

Figure S6 **Illustration of the effect of the transmission-associated rate decline on node height estimation under a molecular clock model.** Each tree depicts the same hypothetical transmission scenario. The transmission event is represented by the transition from black (source) to red (recipient) along the relevant branch. Branch lengths for the left and middle tree are expressed in units of genetic change, whereas they represent time for the tree on the right. The leftmost tree depicts the situation that can be expected under a constant evolutionary rate throughout the evolutionary history, i.e. when the divergence between source and recipient taxa is proportional to their divergence time. The tree in the middle illustrates that transmission of an ancestral variant (the branch part in the source has been evolving at a slower rate) results in a lower then expected divergence. However, under a clock model tips are constrained to be proportional to their sampling time and because of the averaging effect of rate differences under an uncorrelated relaxed clock - rates are drawn from an underlying distribution- the lower divergence between the source and recipient lineages will be reflected in a more recent divergence time for their common ancestor (rightmost tree).
(EPS)

Table S1 **Established transmission intervals.** Samples are indicated by a capital letter to identify the patient, followed by two numbers to indicate the sampling year. If different from the number of sequenced clones, the number of unique sequences is indicated between brackets.
(PDF)

Table S2 **Overview of the conal data.** Samples are indicated by a capital letter to identify the patient, followed by two numbers to indicate the sampling year. If different from the number of sequenced clones, the number of unique sequences is indicated between brackets.
(PDF)

Table S3 **Molecular clock model comparison for the** ***pol*** **and** ***env*** **sequences.** ^1^ PS: path sampling log marginal likelihood estimates. SS: stepping stone sampling log marginal likelihood estimates. Smaller absolute values indicate a better model fit. The similarity between the marginal likelihoods estimated by path sampling (PS) and stepping-stone sampling (SS) suggests adequate convergence properties [35], [36]. ^2^ uced and ucld: uncorrelated relaxed clock models in which the rate of every branch is drawn from an underlying exponential (uced) or lognormal (ucld) distribution.
(PDF)

Table S4 **Demography model comparison results for the** ***pol*** **and** ***env*** **region.** ^1^ PS: path sampling log marginal likelihood estimates. SS: stepping stone sampling log marginal likelihood estimates. Smaller absolute values indicate better model fit. The similarity between the marginal likelihoods estimated by path sampling (PS) and stepping-stone sampling (SS) suggests adequate convergence properties [35], [36].
(PDF)

Table S5 **Fixed effects analyses.** ^1^ Bayes factors (BF) indicate how much the posterior (the result) deviates from the prior (the initial beliefs). In general, BF <3 are considered as absence of support. ^2^ These results refer to the comparison of the within host rate of the transmission chain subjects with the within host rate of the control patients. ^3^ Here, the within host rate estimates of the ‘early’ group (only control subjects) are weighed against the rate estimate of the ‘late’ group (both control and transmission chain patients).
(PDF)

Table S6 **Evolutionary rate estimates and support for the fixed effect in the mixed effects clock model using a flexible coalescent prior.** Whereas the estimates in Table 2 were obtained under the transmission model, the estimates in this table were obtained using the Bayesian skyride model as a tree prior. The mean evolutionary rate and highest posterior density (HPD) intervals are expressed as the number of nucleotide substitutions (10^−3^) per site per year. The Bayes factor (BF) is computed as the posterior odds over the prior odds that the rate for the transmission branches or transmitted lineage branches is smaller than the within host-rate.
(PDF)

Table S7 **Detailed information on the** ***pol*** **within host rate control data sets.** ^1^ median number of time points: 7 (range: 2–12). Median time period covered: 148 (range: 4–350). ^2^ median number of time points: 7 (range: 3–19). Median time period covered: 1170 (range: 434–4352). ^3^ When all samples were taken within the first year after the known or estimated date of infection, the sampled disease stage is labeled ‘early’. When later samples were available, the sampled disease stage is labeled as ‘chronic’. ^4^ All sample dates were, if not yet specified as such, converted to days. Whenever the date was specified as mm-yyyy instead of dd-mm-yyyy, the day was set tot the 15*^th^*. When the sampling date was only specified as a year, the data were not taken into account.
(PDF)

Table S8 **Detailed information on the** ***env*** **within host rate control data sets.** ^1^: median number of time points: 5,5 (range: 2–12). Median time period covered: 184 (range: 4–341). ^2^: median number of time points: 6,5 (range: 3–11). Median time period covered: 1118 (range: 1142–3661). ^3,4^ Determination of the ‘early’ and ‘chronic’ stages as well as the dating were done in the same manner as for the *pol* data. The sample date for the siblings studied by Draenert *et al.* [69] was given as months after infection. Here, the conversion was done by assuming 30 days per month. ^5^: Additional sequences for this patient were available from [70]. ^6^: Additional sequences for this patient were available from [71] and [3]. ^7^: Additional sequences for this patient were available from [71].
(PDF)
