# Supplementary material for: The Genealogical Population Dynamics of HIV-1 in a Large Transmission Chain: Bridging within and among Host Evolutionary Rates
Source: PLoS Comput Biol. 2014 Apr 3;10(4):e1003505. doi: 10.1371/journal.pcbi.1003505 (PMC3974631; doi:10.1371/journal.pcbi.1003505)
Supplement: Table S1 — Established transmission intervals. Samples are indicated by a capital letter to identify the patient, followed by two numbers to indicate the sampling year. If different from the number of sequenced clones, the number of unique sequences is indicated between brackets. (PDF) [file pcbi.1003505.s007.pdf]

Table S1: Established transmission intervals.

	window of transmission
A $\rightarrow$ B†	? - 07/05/90
B $\rightarrow$ A	? - 14/05/90
A $\rightarrow$ F	01/02/95 - 02/08/95
F $\rightarrow$ G	16/01/02 - 16/04/02
B $\rightarrow$ H	29/06/95 - 24/07/95
B $\rightarrow$ I	01/02/93 - 28/04/93
B $\rightarrow$ C	23/09/93 - 10/01/94
C $\rightarrow$ D	16/03/95 - 01/07/95
C $\rightarrow$ L	23/09/93 - 12/03/06
C $\rightarrow$ E	15/06/00 - 01/02/01
E $\rightarrow$ K	01/06/04 - 15/09/04
