# Supplementary material for: The Genealogical Population Dynamics of HIV-1 in a Large Transmission Chain: Bridging within and among Host Evolutionary Rates
Source: PLoS Comput Biol. 2014 Apr 3;10(4):e1003505. doi: 10.1371/journal.pcbi.1003505 (PMC3974631; doi:10.1371/journal.pcbi.1003505)
Supplement: Table S2 — Overview of the conal data. Samples are indicated by a capital letter to identify the patient, followed by two numbers to indicate the sampling year. If different from the number of sequenced clones, the number of unique sequences is indicated between brackets. (PDF) [file pcbi.1003505.s008.pdf]

Table S2: Overview of the conal data.

	sample date	*pol*	*env*	viral load (copies/mL)
A96	03-Jul-1996	12 (11)	3	234.410
A00	25-Sep-2000	10	9	51.152
A05_1	17-Oct-2005	11 (10)	10 (8)	1908
A05_2	7-Nov-2005	10 (8)	7	244
A06	03-Jan-2006	-	8	unknown
B90	07-May-1990	8	8 (6)	unknown
B96	14-Aug-1996	14	11 (9)	622.727
C94	10-Jan-1994	4	16 (15)	unknown
C02	21-01-2002	7	15 (14)	16.785
C03	15-12-2003	10	12 (10)	263.365
D99	16-May-1999	9	12	40.549
D01	02-Apr-2001	10 (9)	16 (13)	64.482
D03	24-Mar-2003	13	15	83.803
E01	21-Feb-2001	11	10 (9)	25.392
E05_1	26-Jan-2005	11 (10)	14	12.071
E05_2	27-Jul-2005	13	7	unknown
E06	22-Feb-2006	14	12 (11)	unknown
F02	17-Apr-2002	11 (10)	19 (13)	66.818
F05	19-Sep-2005	14	1	4.778
G02	16-Apr-2002	13 (4)	10	47.953
H96	15-Jul-1996	13 (11)	6	144.094
H98	25-May-1998	8	11	38.774
I99	06-Oct-1999	10	4 (3)	3.666
K04	30-Sep-2004	13	3	>500.000
L06	24-03-2006	14	7	8.913
TOTAL:		263 (245)	246 (224)	
