# Supplementary material for: The Genealogical Population Dynamics of HIV-1 in a Large Transmission Chain: Bridging within and among Host Evolutionary Rates
Source: PLoS Comput Biol. 2014 Apr 3;10(4):e1003505. doi: 10.1371/journal.pcbi.1003505 (PMC3974631; doi:10.1371/journal.pcbi.1003505)
Supplement: Table S3 — Molecular clock model comparison for the pol and env sequences. 1 PS: path sampling log marginal likelihood estimates. SS: stepping stone sampling log marginal likelihood estimates. Smaller absolute values indicate a better model fit. The similarity between the marginal likelihoods estimated by path sampling (PS) and stepping-stone sampling (SS) suggests adequate convergence properties [35], [36]. 2 uced and ucld: uncorrelated relaxed clock models in which the rate of every branch is drawn from an underlying exponential (uced) or lognormal (ucld) distribution. (PDF) [file pcbi.1003505.s009.pdf]

Table S3: Molecular clock model comparison for the *pol* and *env* sequences

	pol		***env***	
	PS[1]	SS[1]	PS	SS
strict clock	-13717,95	-13722,36	-15564,97	-15569,46
uced[2]	-13691,15	-13693,58	-15531,19	-15534,70
ucld[2]	-13679,69	-13684,53	-15509,46	-15512,79
