# Supplementary material for: The Genealogical Population Dynamics of HIV-1 in a Large Transmission Chain: Bridging within and among Host Evolutionary Rates
Source: PLoS Comput Biol. 2014 Apr 3;10(4):e1003505. doi: 10.1371/journal.pcbi.1003505 (PMC3974631; doi:10.1371/journal.pcbi.1003505)
Supplement: Table S4 — Demography model comparison results for the pol and env region. 1 PS: path sampling log marginal likelihood estimates. SS: stepping stone sampling log marginal likelihood estimates. Smaller absolute values indicate better model fit. The similarity between the marginal likelihoods estimated by path sampling (PS) and stepping-stone sampling (SS) suggests adequate convergence properties [35], [36]. (PDF) [file pcbi.1003505.s010.pdf]

Table S4: Demography model comparison results for the *pol* and *env* region

	pol		***env***	
	PS[1]	SS[1]	PS	SS
constant population size	-13463.87	-13465.11	-15287.30	-15288.20
exponential growth	-13447.09	-13448.04	-15282.72	-15284.04
logistic growth	-13430.93	-13431.56	-15294.40	-15295.89
