# Supplementary material for: The Genealogical Population Dynamics of HIV-1 in a Large Transmission Chain: Bridging within and among Host Evolutionary Rates
Source: PLoS Comput Biol. 2014 Apr 3;10(4):e1003505. doi: 10.1371/journal.pcbi.1003505 (PMC3974631; doi:10.1371/journal.pcbi.1003505)
Supplement: Table S5 — Fixed effects analyses. 1 Bayes factors (BF) indicate how much the posterior (the result) deviates from the prior (the initial beliefs). In general, BF <3 are considered as absence of support. 2 These results refer to the comparison of the within host rate of the transmission chain subjects with the within host rate of the control patients. 3 Here, the within host rate estimates of the ‘early’ group (only control subjects) are weighed against the rate estimate of the ‘late’ group (both control and transmission chain patients). (PDF) [file pcbi.1003505.s011.pdf]

Table S5: Fixed effects analyses

		δ	Bayes factor[1]
therapy impact [2]	*pol*	0.02	0.02
	env	0.01	0.01
rate accelaration[3]	*pol*	0.65	1.89
	env	0.20	0.25
