# Supplementary material for: The Genealogical Population Dynamics of HIV-1 in a Large Transmission Chain: Bridging within and among Host Evolutionary Rates
Source: PLoS Comput Biol. 2014 Apr 3;10(4):e1003505. doi: 10.1371/journal.pcbi.1003505 (PMC3974631; doi:10.1371/journal.pcbi.1003505)
Supplement: Table S6 — Evolutionary rate estimates and support for the fixed effect in the mixed effects clock model using a flexible coalescent prior. Whereas the estimates in Table 2 were obtained under the transmission model, the estimates in this table were obtained using the Bayesian skyride model as a tree prior. The mean evolutionary rate and highest posterior density (HPD) intervals are expressed as the number of nucleotide substitutions (10−3) per site per year. The Bayes factor (BF) is computed as the posterior odds over the prior odds that the rate for the transmission branches or transmitted lineage branches is smaller than the within host-rate. (PDF) [file pcbi.1003505.s012.pdf]

Table S6: Evolutionary rate estimates and support for the fixed effect in the mixed effects clock model using a flexible coalescent prior.

Fixed effects	pol (no DRMs)		env	
	rate	ln BF	rate	ln BF
transmitted lineage branches	2.92 (2.01,3.89)	>6.46	5.86 (4.03,7.78)	3.97
within-host branches	6.26 (5.09,7.66)		8.42 (7.00,10.19)	
