# Supplementary material for: The Genealogical Population Dynamics of HIV-1 in a Large Transmission Chain: Bridging within and among Host Evolutionary Rates
Source: PLoS Comput Biol. 2014 Apr 3;10(4):e1003505. doi: 10.1371/journal.pcbi.1003505 (PMC3974631; doi:10.1371/journal.pcbi.1003505)
Supplement: Table S7 — Detailed information on the pol within host rate control data sets. 1 median number of time points: 7 (range: 2–12). Median time period covered: 148 (range: 4–350). 2 median number of time points: 7 (range: 3–19). Median time period covered: 1170 (range: 434–4352). 3 When all samples were taken within the first year after the known or estimated date of infection, the sampled disease stage is labeled ‘early’. When later samples were available, the sampled disease stage is labeled as ‘chronic’. 4 All sample dates were, if not yet specified as such, converted to days. Whenever the date was specified as mm-yyyy instead of dd-mm-yyyy, the day was set tot the 15th. When the sampling date was only specified as a year, the data were not taken into account. (PDF) [file pcbi.1003505.s013.pdf]

Table S7: Detailed information on the *pol* within host rate control data sets.

***pol*: early[1,3]**

paper	patient ID	# time points	# days between first and last sample[4]
Herbeck *et al.* [7]	11286	3	13
	38051	2	4
	38417	9	218
	71101	8	174
	83747	12	148
	90770	7	194
Kearney *et al.* [8]	1001	2	138
	1002	8	350
	1003	6	120
	1006	7	271

***pol*: chronic[2,3]**

paper	patient ID	# time points	# days between first and last sample
Herbeck *et al.* [7]	55751	4	3376
Kearney *et al.* [8]	1004	9	489
	1005	7	1253
	3021	9	1546
	3024	6	1156
	3036	4	434
	3037	6	910
	3041	6	903
	3062	5	1000
	3077	5	632
	3088	6	995
Kemal *et al.* [10]	WC3	10	4352
Little *et al.* [11]	01-0180	18	1210
	01-0449	17	1495
	01-0507	12	1442
	01-0512	19	1439
	01-0559	11	1170
	01-0575	15	1106
	01-0629	12	1169
Liu *et al.* [12]	PIC1362	3	1249

References

[1] Katharine J Bar, Chun-yen Tsao, Shilpa S Iyer, Julie M Decker, Yongping Yang, Mattia Bonsignori, Xi Chen, Kwan-Ki Hwang, David C Montefiori, Hua-Xin Liao, Peter Hraber, William Fischer, Hui Li, Shuyi Wang, Sarah Sterrett, Brandon F Keele, Vitaly V Ganusov, Alan S Perelson, Bette T Korber, Ivelin Georgiev, Jason S McLellan, Jeffrey W Pavlicek, Feng Gao, Barton F Haynes, Beatrice H Hahn, Peter D Kwong, and George M Shaw. Early low-titer neutralizing antibodies impede HIV-1 replication and select for virus escape. *PLoS Pathog*, 8(5):e1002721, 2012.

[2] Samatchaya Boonchawalit, Duangrat Jullaksorn, Jiraporn Uttiyoung, Amara Yowang, Nongkran Krathong, Sununta Chautrakul, Akifumi Yamashita, Kazuyoshi Ikuta, Amornsak Roobsoong, Sangkom Kanitvittaya, Pathom Sawanpanyalert, and Masanori Kameoka. Molecular evolution of HIV-1 CRF01_AE Env in Thai patients. *PLoS One*, 6(11):e27098, 2011.

[3] Mia Coetzer, Rebecca Nedellec, Tonie Cilliers, Tammy Meyers, Lynn Morris, and Donald E Mosier. Extreme genetic divergence is required for coreceptor switching in HIV-1 subtype C. *J Acquir Immune Defic Syndr*, 56(1):9–15, Jan 2011.

[4] Julie M Decker, Frederic Bibollet-Ruche, Xiping Wei, Shuyi Wang, David N Levy, Wenquan Wang, Eric Delaporte, Martine Peeters, Cynthia A Derdeyn, Susan Allen, Eric Hunter, Michael S Saag, James A Hoxie, Beatrice H Hahn, Peter D Kwong, James E Robinson, and George M Shaw. Antigenic conservation and immunogenicity of the HIV coreceptor binding site. *J Exp Med*, 201(9):1407–19, May 2005.

[5] Rika Draenert, Todd M Allen, Yang Liu, Terri Wrin, Colombe Chappey, Cori L Verrill, Guillem Sirera, Robert L Eldridge, Matthew P Lahaie, Lidia Ruiz, Bonaventura Clotet, Christos J Petropoulos, Bruce D Walker, and Javier Martinez-Picado. Constraints on HIV-1 evolution and immunodominance revealed in monozygotic adult twins infected with the same virus. *J Exp Med*, 203(3):529–39, Mar 2006.

[6] Ceiridwen J Edwards, Marc A Suchard, Philippe Lemey, John J Welch, Ian Barnes, Tara L Fulton, Ross Barnett, Tamsin C O'Connell, Peter Coxon, Nigel Monaghan, Cristina E Valdiosera, Eline D Lorenzen, Eske Willerslev, Gennady F Baryshnikov, Andrew Rambaut, Mark G Thomas, Daniel G Bradley, and Beth Shapiro. Ancient hybridization and an Irish origin for the modern polar bear matriline. *Curr Biol*, 21(15):1251–8, Aug 2011.

[7] Joshua T Herbeck, Morgane Rolland, Yi Liu, Sherry McLaughlin, John McNevin, Hong Zhao, Kim Wong, Julia N Stoddard, Dana Raugi, Stephanie Sorensen, Indira Genowati, Brian Birditt, Angela McKay, Kurt Diem, Brandon S Maust, Wenjie Deng,

Ann C Collier, Joanne D Stekler, M Juliana McElrath, and James I Mullins. Demographic processes affect HIV-1 evolution in primary infection before the onset of selective processes. *J Virol*, 85(15):7523–34, Aug 2011.

[8] M Kearney, F Maldarelli, W Shao, J B Margolick, E S Daar, J W Mellors, V Rao, J M Coffin, and S Palmer. Human immunodeficiency virus type 1 population genetics and adaptation in newly infected individuals. *J Virol*, 83(6):2715–27, Mar 2009.

[9] Brandon F Keele, Elena E Giorgi, Jesus F Salazar-Gonzalez, Julie M Decker, Kimmy T Pham, Maria G Salazar, Chuanxi Sun, Truman Grayson, Shuyi Wang, Hui Li, Xiping Wei, Chunlai Jiang, Jennifer L Kirchherr, Feng Gao, Jeffery A Anderson, Li-Hua Ping, Ronald Swanstrom, Georgia D Tomaras, William A Blattner, Paul A Goepfert, J Michael Kilby, Michael S Saag, Eric L Delwart, Michael P Busch, Myron S Cohen, David C Montefiori, Barton F Haynes, Brian Gaschen, Gayathri S Athreya, Ha Y Lee, Natasha Wood, Cathal Seoighe, Alan S Perelson, Tanmoy Bhattacharya, Bette T Korber, Beatrice H Hahn, and George M Shaw. Identification and characterization of transmitted and early founder virus envelopes in primary HIV-1 infection. *Proc Natl Acad Sci U S A*, 105(21):7552–7, May 2008.

[10] Kimdar Sherefa Kemal, Tara Beattie, Tao Dong, Barbara Weiser, Rupert Kaul, Carla Kuiken, Julian Sutton, Dorothy Lang, Hongbing Yang, Yan Chun Peng, Ronald Collman, Sean Philpott, Sarah Rowland-Jones, and Harold Burger. Transition from long-term nonprogression to HIV-1 disease associated with escape from cellular immune control. *J Acquir Immune Defic Syndr*, 48(2):119–26, Jun 2008.

[11] Susan J Little, Simon D W Frost, Joseph K Wong, Davey M Smith, Sergei L Kosakovsky Pond, Caroline C Ignacio, Neil T Parkin, Christos J Petropoulos, and Douglas D Richman. Persistence of transmitted drug resistance among subjects with primary human immunodeficiency virus infection. *J Virol*, 82(11):5510–8, Jun 2008.

[12] Yi Liu, John McNevin, Jianhong Cao, Hong Zhao, Indira Genowati, Kim Wong, Sherry McLaughlin, Matthew D McSweyn, Kurt Diem, Claire E Stevens, Janine Maenza, Hongxia He, David C Nickle, Daniel Shriner, Sarah E Holte, Ann C Collier, Lawrence Corey, M Juliana McElrath, and James I Mullins. Selection on the human immunodeficiency virus type 1 proteome following primary infection. *J Virol*, 80(19):9519–29, Oct 2006.

[13] Madhumita Mahalanabis, Pushpa Jayaraman, Toshiyuki Miura, Florencia Pereyra, E Michael Chester, Barbra Richardson, Bruce Walker, and Nancy L Haigwood. Continuous viral escape and selection by autologous neutralizing antibodies in drug-naive human immunodeficiency virus controllers. *J Virol*, 83(2):662–72, Jan 2009.

[14] Helena Skar, Ryan N Gutenkunst, Karin Wilbe Ramsay, Annette Alaeus, Jan Albert, and Thomas Leitner. Daily sampling of an HIV-1 patient with slowly progressing

disease displays persistence of multiple env subpopulations consistent with neutrality. *PLoS One*, 6(8):e21747, 2011.

[15] Marit J van Gils, Evelien M Bunnik, Judith A Burger, Yodit Jacob, Becky Schweighardt, Terri Wrin, and Hanneke Schuitemaker. Rapid escape from preserved cross-reactive neutralizing humoral immunity without loss of viral fitness in HIV-1-infected progressors and long-term nonprogressors. *J Virol*, 84(7):3576–85, Apr 2010.

[16] Xiping Wei, Julie M Decker, Shuyi Wang, Huxiong Hui, John C Kappes, Xiaoyun Wu, Jesus F Salazar-Gonzalez, Maria G Salazar, J Michael Kilby, Michael S Saag, Natalia L Komarova, Martin A Nowak, Beatrice H Hahn, Peter D Kwong, and George M Shaw. Antibody neutralization and escape by HIV-1. *Nature*, 422(6929):307–12, Mar 2003.

[17] Xueling Wu, Charlene Wang, Sijy O'Dell, Yuxing Li, Brandon F Keele, Zhongjia Yang, Hiromi Imamichi, Nicole Doria-Rose, James A Hoxie, Mark Connors, George M Shaw, Richard T Wyatt, and John R Mascola. Selection pressure on HIV-1 envelope by broadly neutralizing antibodies to the conserved CD4-binding site. *J Virol*, 86(10):5844–56, May 2012.
